# Perspective: The Place of Milk, Yogurt, and Cheese in Sustainable Healthy Diets in France

**DOI:** 10.1016/j.cdnut.2026.109538

**Published:** 2026-09-19

**Authors:** Adam Drewnowski, Florent Vieux, Matthieu Maillot

**Affiliations:** 1 Center for Public Health Nutrition, University of Washington, Seattle, WA, United States; 2 MS-Nutrition, Marseille, France

**Keywords:** dairy foods, nutrient density, affordability, greenhouse gas emissions (GHGE), sustainable diets, CIQUAL, INCA3, Agribalyse

## Abstract

The 4 pillars of sustainable healthy diets are health, economics, society, and the environment. The place of milk, yogurt, and cheese in the sustainability framework was examined using publicly available government agency databases from France. Nutrient composition data came from CIQUAL (Centre d’Information sur la Qualité des Aliments). Data on greenhouse gas emissions (GHGE) and total environmental impact of foods came from Agribalyse. Data on food costs came from published reports. Dietary intakes came from the INCA 3 study (Étude Individuelle Nationale des Consommations Alimentaires) of French children (3–17 y, *n* = 1775) and adults (18–79 y, *n* = 2121). Two version of the Nutrient Rich Food Index (NRF6.3 and NRF9.3) were applied to protein foods and to milk, yogurt, and cheese. The NRF model is composed of 2 subscores NR and LIM. The protein component of NR was corrected for PDCAAS (Protein Digestibility-Corrected Amino Acid Score. The LIM component was based on saturated fat, added sugar, and sodium. Diet quality metrics were based on the ratio of nutrients to calories. Nutrient density scores for dairy spanned a wide range from skim milk to blue cheese. Based on Agribalyse data for France, milk and dairy were associated with lower GHGE and environmental cost than red meat and were closer to eggs and some plant proteins. The cost in euros per kilogram and per 100 kcal was lower than red meat but above eggs and legumes. Diet-level analyses showed that milk, yogurt, and cheese provided more nutrients than calories to the total diet, meeting the definition of nutrient-rich foods. Milk, yogurt, and especially cheese, with its rich culinary history, are integral components of the French diet. Dairy foods provide affordable nutrient density and have a relatively low impact on the environment.

## Introduction

Based on a definition adopted by the Food and Agriculture Organization of the United Nations (FAO), the 4 pillars of sustainability are health, economics, society, and the environment [1]. Sustainable diets are nutrient-rich, affordable, socially and culturally acceptable, and consistent with planetary boundaries [2,3]. Although dairy foods are an important and culturally significant component of human nutrition, they are said to have a high environmental cost [4,5]. With the current emphasis on reducing the ratio of animal-to-plant proteins in the global diet [[6], [7], [8]], the place of milk and dairy in the sustainability framework needs to be examined in more detail.

Milk, yogurt, and especially cheese, occupy a special place in the French culinary tradition, reflecting centuries of regional food heritage and agricultural practices [9]. The common belief is that the per capita consumption of yogurt and cheese in France is particularly high. At least, that is suggested by agricultural outlook reports from the FAO [10] and by marketing sources. Cheese availability from food balance sheets was reported to be ∼23.7 kg/person/y [11], which would translate to ∼65 g/d, placing France among the highest-ranking countries globally.

There are relatively few studies of the place of milk, yogurt, and cheese in sustainable healthy diets in France. Analyses to support the present Perspective had to be based on publicly available databases developed and maintained by French government agencies. The main challenge was to assemble contest-specific data on nutrient composition, monetary costs, environmental impact, and consumption trends. Whereas data on nutrient composition of foods and on environmental costs are readily available, epidemiologic data on consumption trends across population strata in France are scarce [12].

Past analyses of the 4 pillars of the sustainability framework as applied to dairy foods were often based on publicly available data from the United States [13,14]. Nutrient density estimates showed that milk and dairy contributed only 12% of dietary energy but much higher proportions of calcium, riboflavin (B2), vitamin A, and vitamin D to the United States diet [9], which made them nutrient-rich by definition [15]. Dairy is also an important source of protein. Analyses of the USDA protein foods group showed that milk, yogurt, and cheese provided ≥7 g protein per serving, equivalent to 1 oz (30 g) of meat [14]. Recent data from Scotland confirmed that dairy products were important sources of calcium, iodine, and vitamin A [16].

Analyses of national food prices in the United States showed that milk and dairy foods were by far the lowest-cost sources of dietary calcium and an affordable source of high-quality protein [17]. Similar observations had been made in France [18]. Further, analyses of dietary patterns in the United States and in France showed wide acceptance of dairy foods across population groups [15] and explored the carbon footprint of self-selected alternative diets [19,20]. Analyses of environmental impact showed that milk and dairy foods were associated with lower greenhouse gas emissions (GHGE) relative to ruminant meat [[19], [20]].

The place of milk, yogurt, and cheese in sustainable healthy diets needed to be confirmed using similar analyses based on data from outside the United States. This Perspective was supported by novel analyses of context-specific data from France.

## Databases and Analytical Methods

Analyses were conducted at the food and diet level. Food-level data included nutrient composition, estimated cost, and environmental impact of foods. Those data came from publicly available French government sources. Dietary intakes came from the most recent nationally representative dietary survey [Étude Individuelle Nationale des Consommations Alimentaires (INCA3)] of children and adults, conducted in 2014.

### The ANSES/CIQUAL nutrient composition database

Nutrient composition data came from the French Agency for Food, Environmental and Occupational Health & Safety [Agence Nationale de Sécurité Sanitaire de l’Alimentation, de l’Environnement et du Travail (ANSES)] [21]. The ANSES/CIQUAL nutrient composition database developed by Centre d’Information sur la Qualité des Aliments (CIQUAL) is the core resource for research, food labeling, and public health policy initiatives in France. The 2025 version of ANSES/CIQUAL database (CIQUAL for short) lists 3484 foods, including both raw and processed foods, that are representative of the French diet. Among the protein foods were meat, poultry and fish, eggs, milk and dairy, pulses, legumes, and nuts and seeds. Data were available for energy, macronutrients, fiber, and 27 vitamins and minerals per 100 g [21]. The added sugar content of foods, unavailable in CIQUAL, was estimated following a previously published methodology [22].

The present analyses were restricted to milk and dairy products along with foods in the protein food group. For comparison purposes, the What We Eat in America (WWEIA) protein food group includes red meat, poultry and seafood, processed meat, eggs, and plant protein sources, including legumes, nuts, and seeds [14]. Foods in the CIQUAL database were aggregated into food groups, subgroups, and categories that paralleled the WWEIA scheme.

Vieux et al. [19] used a comparable scheme to assign CIQUAL foods into 20 categories. Mixed dishes, condiments, alcohols, water, and infant foods were excluded. Foods and beverages (*n* = 1809) were divided into meats, eggs and dairy, and plant-based foods. The selected categories of interest were ruminant meat (beef), other meats, processed meat, seafood, and eggs and dairy, along with cereals, legumes, nuts and seeds, and vegetables and fruit. Portion sizes came from Vieux et al. [19].

### GHGE values from Agribalyse

Data on the environmental impact of foods from farm production to the consumer came from Agribalyse V3.1, the official lifecycle assessment [lifecycle analysis (LCA)] database of food items consumed in France [23]. The Agribalyse database belongs to the French Agency for Ecological Transition or Agence de la transition écologique and to Institut national de recherche pour l’agriculture, l’alimentation et l’environnement (INRAE or National Research Institute for Agriculture, Food, and Environment). Two indicators were selected for the present analyses: GHGE (in kg carbon dioxide eq) and the composite environmental impact score product environmental footprint (PEF), which is a weighted mean score of the 16 environmental metrics presented in Agribalyse V3.1. Foods from Agribalyse V3.1 had been matched to INCA3 food consumption data in a previous study [19].

### Food prices for selected food categories

Data on the monetary cost of 20 food categories as consumed (per kilogram, per 100 kcal, and per serving) came from published supplemental data files for a previous study [19]. Food prices were based on food purchases of 6565 French households of the 2014 Kantar Worldpanel, with each food purchased matched to the closest food in the ANSES/CIQUAL database. The food prices were not corrected for inflation. Details can be found in the Methods section of [19]. The 2014 food prices were corrected for preparation and loss using standard procedures to arrive at a cost per 100 g edible portion [19].

### The Nutrient Rich Food Index

The Nutrient Rich Food Index (NRF) is a well-established nutrient profiling model used to calculate the overall nutrient density of foods [[24], [25], [26]]. The positive Nutrient Rich NR*n* subscore is based on a variable number *n* of nutrients to encourage, and the negative LIM subscore is based on 3 nutrients to limit. Nutrients to encourage are protein, fiber, and a variety of vitamins and minerals. The NRF9.3 (standard version) uses protein, fiber, calcium, iron, potassium, magnesium, vitamin A, vitamin C, and vitamin D [[24], [25], [26]]. Different versions of the NRF have been developed for specific food categories such as grains [27], fruits [28], herbs and spices [29], and seafood [30]. There is also an NRF score developed to assess the Swedish food supply [31].

The present version was a modified Nutrient Rich Food Index (NRF6.3), where the positive NR6 subscore was composed of 6 nutrients to encourage: protein, calcium, iron, vitamin B2, vitamin A, and fiber. Protein content was adjusted using the PDCAAS [32]. Whereas the PDCAAS value for milk and dairy is 1.0, values for plant proteins from grains, pulses, and nuts can be much lower [33]. Percent daily values (%DV) were calculated per 100 kcal and were capped at 100% [[24], [25], [26]]. Although Digestible Indispensable Amino Acid Score (DIAAS) values are currently preferred by the FAO, DIAAS values for a wide range of foods are not yet available.

The negative LIM subscore is typically composed of the same 3 nutrients to limit: saturated fat, added sugar, and sodium. Studies have suggested that saturated fats from different food sources may have a differential impact on health outcomes, depending on the food matrix [34,35]. For the present analyses, we used the original LIM subscore based on saturated fat, added sugar, and sodium. However, there are reasons to explore ways to make existing nutrient profiling models more sensitive to the quality of dietary fats. The final NRFn.3 score is calculated as NRn − LIM, that is, the sum of %DV for n nutrients to encourage minus the sum of %DV for the 3 nutrients to limit. The current nutrient standards for France, as developed by national agencies, were taken from a previous article [19]. Nutrient profiling was conducted using SPSS version 31.

### Dietary intakes from national survey INCA3 (Individual Nutrition Survey 2014)

Dietary intake data for milk, yogurt, and cheese came from the latest French Individual and National Food Consumption (INCA3) Survey 2014–2015, a nationally representative cross-sectional survey carried out by ANSES (French Agency for Food, Environmental and Occupational Health and Safety) in 2014–2015 [36]. The INCA studies, equivalent to the United States National Health and Nutrition Examination Survey (NHANES), are the foundation of nutrition surveillance in France. The total INCA3 cohort was 4114 children and adults living in mainland France and was drawn by stratified random sampling from the national census [36]. Of these, there were 3982 participants with 3 d of dietary recalls and 132 with 2 d of dietary recalls. Children from 0 to 3 y were excluded, leaving a sample size of 1775 children. The final analytical sample was 1775 children (3–17 y) and 2121 adults (18–79 y). Age groups for children were 4 to 6 y, 7 to 10 y, 11 to 14 y, and 15 to 17 y, and for adults 18 to 44 y, 45 to 64 y, and >65 y. Socioeconomic status was divided into low, medium, and high, and a 4th class, labeled as not working. Income per consumption unit (CU) was categorized as <900 €/mo/CU, 900 to 1340 €/mo/CU, 1340 to 1850 €/mo/CU, ≥1850 €/mo/CU. Participants completed 3 nonconsecutive 24-h dietary recalls for 2 weekdays and 1 weekend day spread over 3 wk. One limitation was that dietary intakes of young children were completed by parents or caregivers. Each food was described in terms of preparation and quantity consumed. Nutrient composition data came from the ANSES/CIQUAL 2016 database).

All analyses accounted for the complex survey design of INCA3 data and were representative of the French metropolitan population. Estimates based on INCA3 data were calculated using individual weighting factors provided by ANSES to consider French metropolitan representativeness. As requested by ANSES, children and adults’ subsamples were treated separately. Data analyses used R software version 4.4.

## Results

The present Perspective is supported by combined data from food composition tables, context-specific data on the environmental cost of food production, national food prices, and dietary trends. All analyses were based on publicly available data from French government agencies. These were used to support the following views.

### Milk, yogurt, and cheese are nutrient-rich foods

Analyses of the CIQUAL 2025 database focused on the protein content of foods. Meat, milk, and eggs had more digestible protein per 100 g and per 100 kcal than plant-based proteins such as legumes, nuts, and seeds. Table 1 shows energy density (ED) in kcal/100 g, protein content in g/100 g, protein content in g/100 kcal, and calcium content in mg/100 g by food category. Protein values were corrected for PDCAAS. Dairy products ranged in protein content from ∼4 g/100 g for milk and yogurt to >25 g/100 g for some cheeses. Meat, poultry, fish, and seafood had an average protein content of >20 g protein per 100 g. Fish and seafood, raw and cooked, provided the most protein per 100 kcal, followed by raw meat. Plant-based beverages were poor protein sources per 100 kcal. Cheese and dairy products had the highest calcium content per 100 g, followed by seafood and nuts and seeds. These data are consistent with the corresponding findings in the United States [37].

**TABLE 1:** Energy density (kcal/100 g), protein content (g/100 g and g/100 kcal) and calcium content (mg/100 g) by CIQUAL food category

CIQUAL food categories	*N*	Energy density, kcal/100 g		Protein∗ g/100 g		Protein∗ g/100 kcal		Calcium mg/100 g	
		Mean	SEM	Mean	SEM	Mean	SEM	Mean	SEM
Cheese and similar	153	316.9	5.3	20.06	0.5	6.6	0.1	475.57	24
Dairy beverages	8	60.6	3.2	2.56	0.3	4.16	0.4	95.71	7.2
Dairy milk	13	49.7	3	3.4	0.0	7.18	0.5	119.79	0.3
Dairy products	98	83.1	3	4.83	0.2	6.71	0.4	135.52	2.5
Eggs	22	195.6	26.9	13.37	1.1	8.38	1.0	72.24	13.5
Fish products	75	204.4	8.8	17.15	0.8	9.53	0.6	68.47	12.9
Fish (cooked)	57	137.8	6.6	21.34	0.4	17.07	0.6	32.52	4.2
Fish (raw)	108	113	5.8	17.72	0.2	17.49	0.4	28.35	2.4
Legumes	26	108.9	4.6	5.76	0.2	5.41	0.2	55.01	6
Meat (cooked)	127	185.3	4	25.92	0.4	14.6	0.3	12.9	0.6
Meat (processed)	164	286.9	8.1	16.37	0.4	7.09	0.4	20.85	1.9
Meat (raw)	159	165.7	6	18.4	0.2	12.75	0.4	10.95	0.6
Nuts and seeds	53	567	21.8	9.35	0.9	1.65	0.3	127.72	24.6
Plant-based beverages	15	43.7	3.4	1.31	0.3	2.88	0.7	30.36	7.9
Seafood (cooked)	30	103.7	4	19.27	0.7	18.86	0.5	122.44	38.4
Seafood (raw)	24	82.3	3.5	14.91	0.8	18.06	0.6	56.71	10.8
Soy products	7	136.9	14.2	12.41	2.4	9.06	1.5	56.71	14.0

Data are means and SEM.

∗Protein content after PDCAAS correction.

Abbreviations: CIQUAL, Centre d’Information sur la Qualité des Aliments; PDCAAS, protein digestibility-corrected amino acid score.

Further analyses disaggregated CIQUAL food groups into more granular food categories to better show the placement of milk, yogurt, and cheese relative to the protein food group. Figure 1 shows scatterplots of protein content (g/100 kcal) against ED in kcal/100 g. Seafood provided the most protein per 100 kcal, followed by fish, poultry, red meat, and organ meats. On the ED scale (*y*-axis), milk and yogurt were placed low, cheese was intermediate (300–400 kcal/100g), and nuts and seeds had the highest ED. The size of the circle reflected %DV per 100 kcal for different nutrients that were a part of the composite NRF6.3 nutrient density score. Figure 1A shows that cheeses (hard cheese, blue cheese, soft cheese, and unprocessed cheese) were high in calcium. Figure 1B shows that organ meats, seafood, and red meat were high in iron, whereas milk and dairy products were not. However, reduced and low-fat milk had the highest %DV for vitamin B2 (Figure 1C).

**FIGURE 1:**
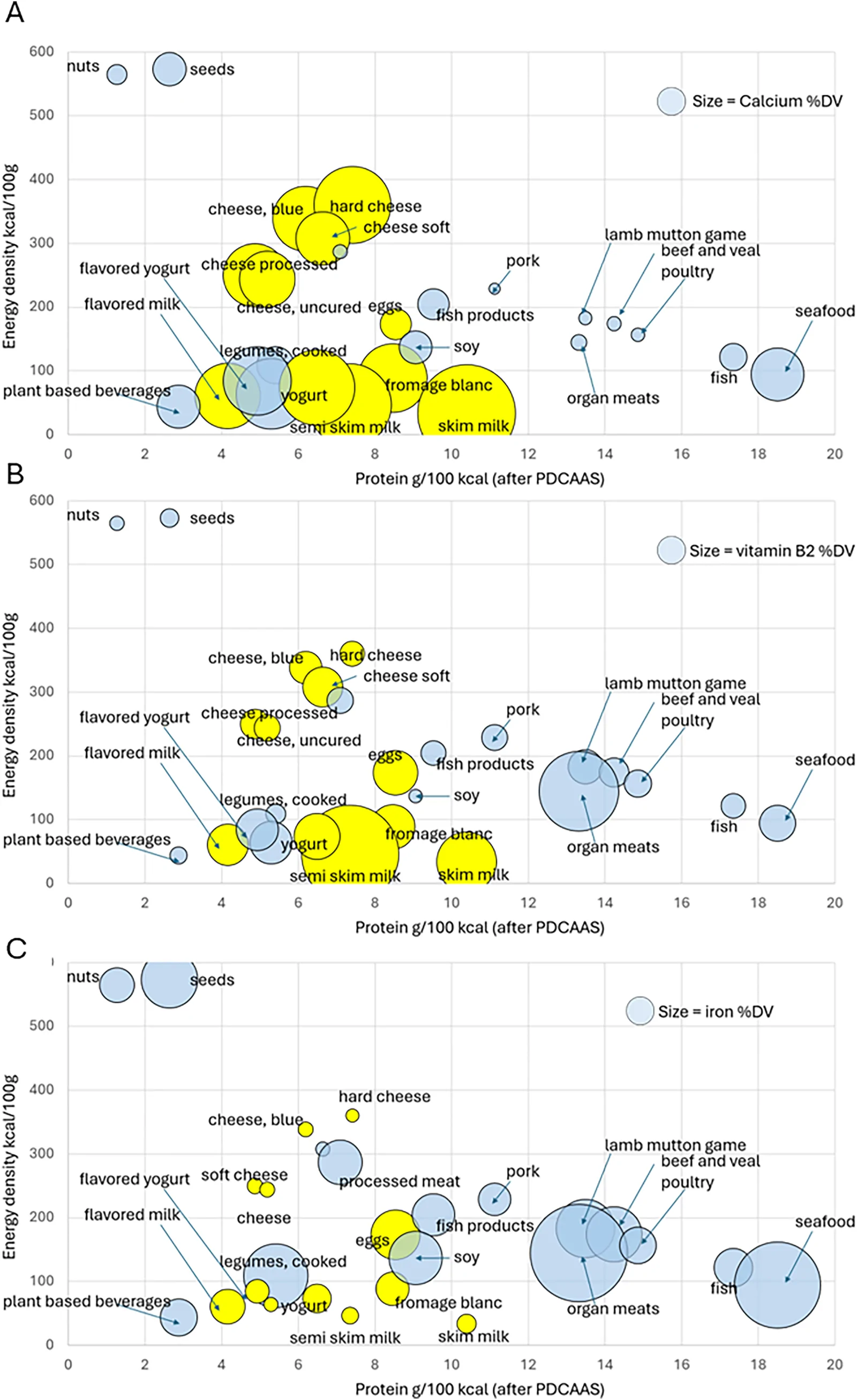
Protein content (g/100 kcal) plotted against energy density (kcal/100 g) for selected ANSES/CIQUAL food categories. Size of bubble reflects percent daily value (%DV) per 100 kcal for calcium (A), iron (B), and vitamin B2 (C). PDCAAS, protein digestibility-corrected amino acid score.

The positive subscore NR6 of the composite NRF6.3 score used protein, fiber, calcium, iron, vitamin B2, and vitamin A as the nutrients to encourage. Saturated fat, added sugar, and sodium were the nutrients to limit. Figure 2A shows that the foods with the highest NRF6.3 scores were organ meats (liver), reduced-fat and skim milk, seafood, and legumes. These were followed by fish, eggs, and then red meat and poultry. Processed meats had the lowest nutrient density scores, largely because of sodium and saturated fat. Position on the vertical axis (ED in kcal/100 g) shows that milks and yogurts were low–energy-density foods, whereas cheeses were higher–energy-density foods.

**FIGURE 2:**
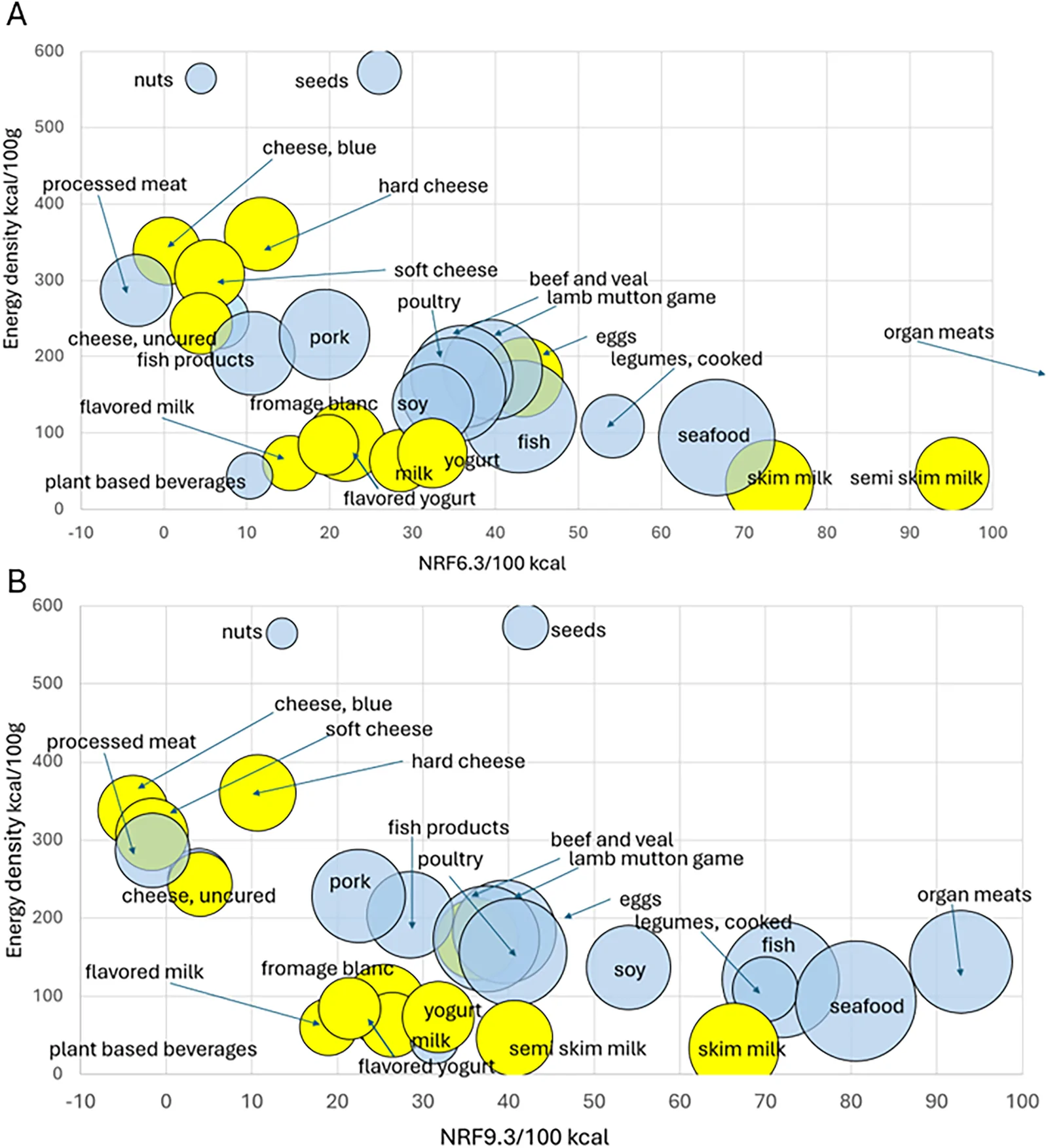
Mean Nutrient Rich Food Index (NRF)6.3 nutrient density scores (A) and mean NRF9.3 nutrient density scores (B) plotted against energy density by food category. The size of the bubble is protein percent daily value per 100 kcal.

The positive subscore NR9 of the full NRF9.3 score used protein, fiber, calcium, iron, potassium, magnesium, and vitamins A, C, and D as the nutrients to encourage. Saturated fat, added sugar, and sodium were the nutrients to limit. Figure 2B shows that the foods with the highest NRF9.3 scores were organ meats (liver), seafood, fish, legumes, and skim milk. These were followed by soy, reduced-fat milk, eggs, yogurt, and then red meat and poultry. Processed meats had the lowest nutrient density scores, largely because of sodium and saturated fat. Position on the vertical axis (ED in kcal/100 g) shows that milks and yogurts were low–energy-density foods, whereas cheeses were higher–energy-density foods. The correlation between NRF6.3 and NRF9.3 scores was 0.805 (*P* < 0.001).

The size of the circle in Figures 2A and 2B is proportional to the %DV of protein per 100 kcal. As noted before, seafood, fish, and meat had the most protein per 100 kcal. Composite nutrient density metrics show that milk and dairy provide more nutrients per calorie than do many alternative foods.

### Milk and dairy provide affordable nutrient density

Dairy products are frequently cited as being among the lowest-cost sources of calcium and high-quality protein [17,25,37]. The present cost data came from supplemental data files for a previous publication [19] that was based on Kantar Worldpanel data for selected retail food prices in France [19]. Figure 3, based on supplemental data from Vieux et al. [19], shows that dairy products cost less per 100 g of product and also cost less per 50 g protein compared with seafood, meat, and poultry. Consistent with other observations [14,25], eggs and dairy foods provided 50 g high-quality animal protein at the lowest cost. Eggs were the lowest-cost source of animal protein. Legumes were the lowest-cost protein source of all.

**FIGURE 3:**
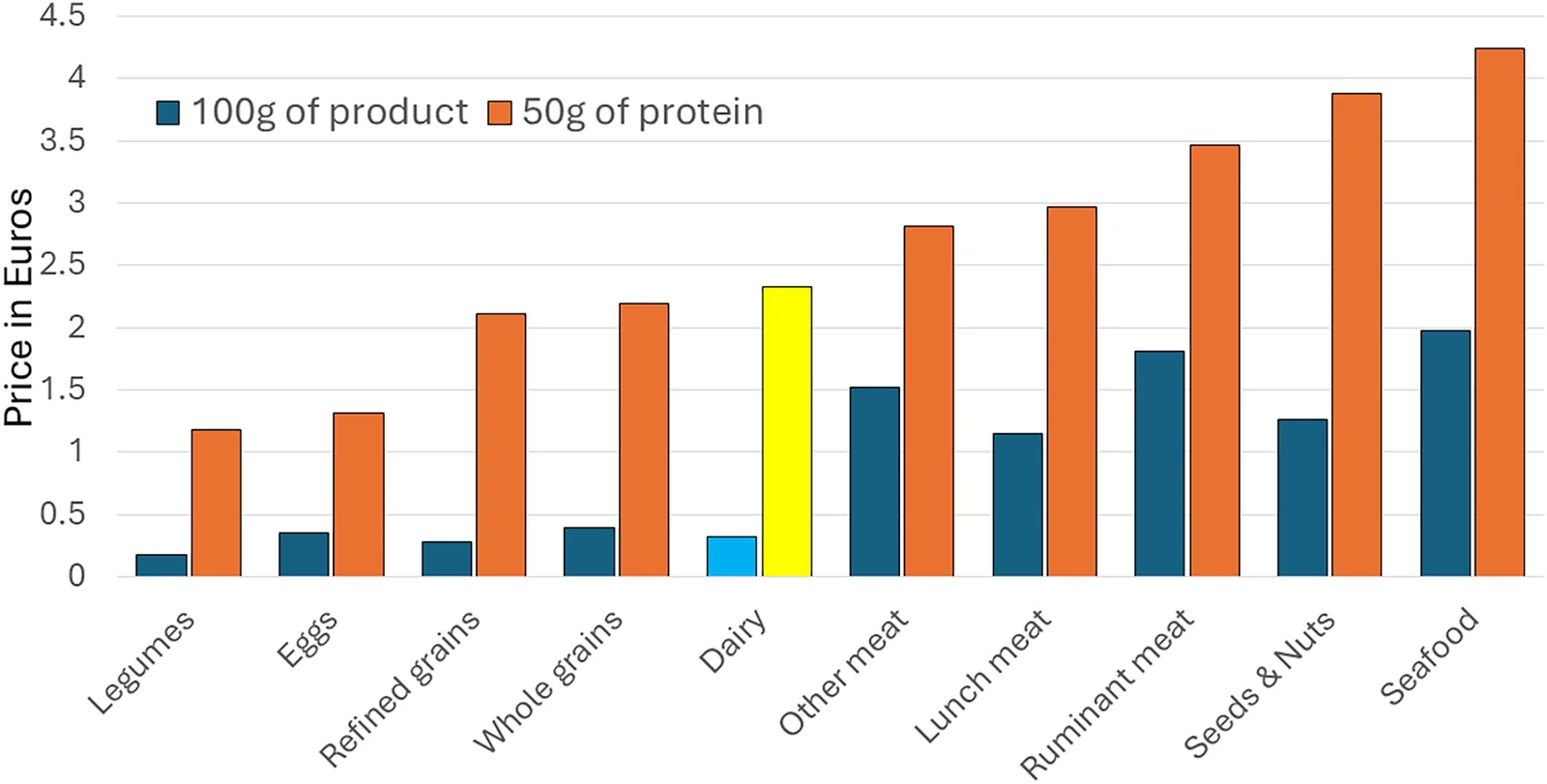
Prices in euros per 100 g product and per 50 g protein. Plot based on data from the study by Vieux et al. [19]. Protein not corrected for protein digestibility-corrected amino acid score.

### Milk and dairy foods have low GHGE

The 2 environmental indicators from the Agribalyse database were GHGE and the PEF. Figure 4A is a log-log scatterplot of mean prices in euros per kilogram and GHGE per kg food by category [19]. The size of the circle is the PEF/kg. Figure 4B is a log-log scatterplot of prices in euros per 100 kcal and GHGE per 100 kcal of food also by category [19]. The size of the circle is the PEF/100 kcal. All data came from supplemental files for a previous publication [19].

**FIGURE 4:**
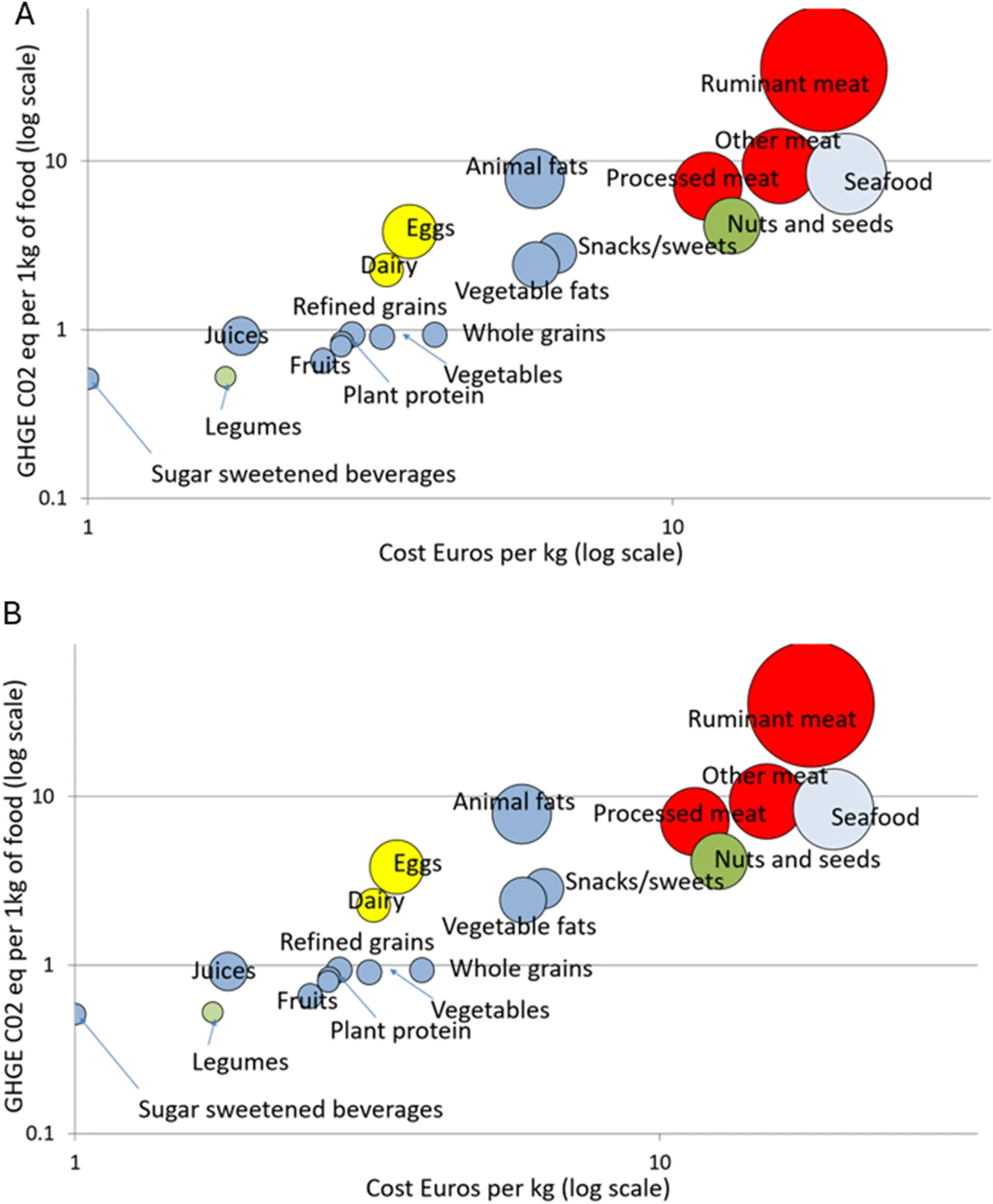
Median costs in euros/kg plotted against greenhouse gas emissions (GHGE) (CO_2_ eq/kg) (A) and median costs in euros/100 kcal plotted against GHGE (CO_2_ eq/100 kcal) (B). The size of the circle is product environmental footprint per kg (A) and per kcal (B). Plot based on data from the study by Vieux et al. [19]. CO_2_, carbon dioxide.

As expected, the highest cost per 100 g was for seafood, beef (ruminant meat), other meat, and nuts and seeds. The lowest cost was for sugar-sweetened beverages. Eggs and dairy were closer in cost per kilogram to grains, vegetables, and fruit than to animal proteins. Costs calculated per 100 kcal only made these differences more apparent. Eggs and dairy were closer to legumes and grains than to meat, poultry, and fish.

Figure 4 shows GHGE values (per kg and per 100 kcal) on the vertical axis. Red meat had higher GHGE values than seafood and nuts and seeds. The GHGE associated with dairy were well below that of ruminant meat and were closer to that of eggs, chicken, and beans. The size of the circle reflects the overall PEF. Milk and dairy were animal-based foods with the lowest environmental impact. Eggs and some plant-based protein sources (legumes and soy products) had both low cost and low environmental impact.

### The social component—food patterns in France

The wide acceptance of milk, yogurt, and cheese across diverse age groups in France was supported by analyses of the INCA3 dietary surveys. About 98% of INCA3 participants consumed milk or dairy products over the 3 d of INCA3 data collection. Figure 5 shows percent daily calories from different types of milk, yogurt, and cheese by age group. Skim milk was consumed more than whole milk, and flavored milk was consumed almost not at all. Sweetened yogurt was consumed more than plain yogurt by children and young adults. Older adults consumed more unsweetened and plain yogurt and harder and soft cheese. The consumption of cheese, both hard and soft, increased with age, especially after the age of 65 y.

**FIGURE 5:**
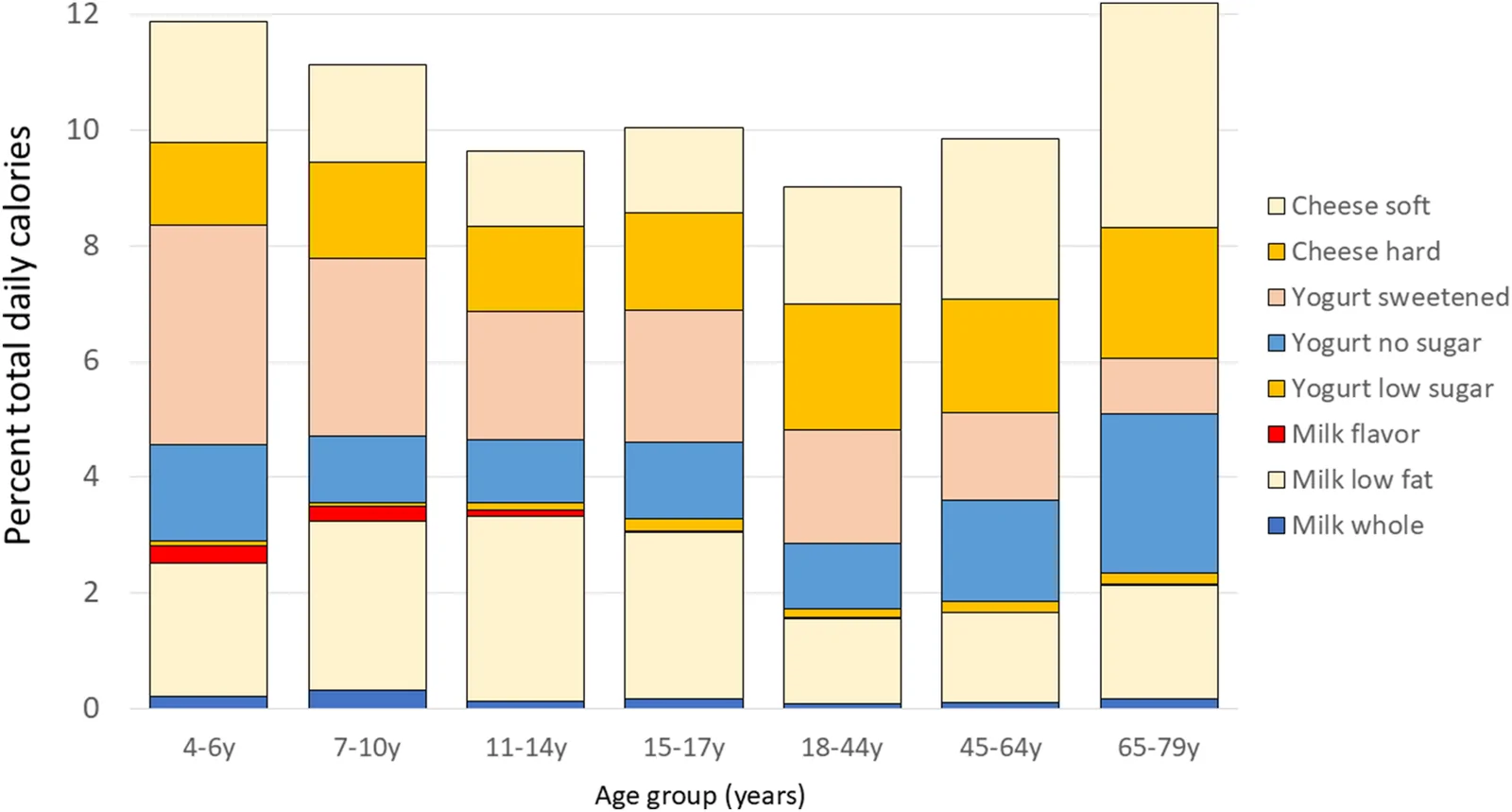
Percent contribution of different types of milk, yogurt, and cheese to total daily calories by age group.

### Diet-level metrics of nutrient density using INCA3 data

Milk, yogurt, and cheese delivered multiple nutrients for relatively few calories. Figure 6 shows the contribution of milk, yogurt, and cheese to energy and nutrient intakes in the INCA3 study for children (Figure 6A) and for adults (Figure 6B). Energy from milk and dairy foods represented <10% of total daily intake between the ages of 11 y and 64 y and ∼12% for <11 y and >65 y age groups. For children, dairy products contributed ∼11% (SD = 6.8, SEM = 0.3) of energy but 36.3% (SD = 19.4, SEM = 0.8) of calcium, 33.1% (SD = 20.7, SEM = 0.8) of vitamin D, 22.8% (SD = 13.6, SEM = 0.6) of phosphorus, 21.9% (SD = 15.2, SEM = 0.6) of vitamin B12, 16.9% (SD = 11.1, SEM = 0.4) of zinc, and 16.1% (SD = 12.3, SEM = 0.4) of vitamin A. Dairy products also contributed ∼20% (SD = 13.1, SEM = 0.5) of saturated fat. For adults, dairy products contributed ∼10% (SD = 6.3, SEM = 0.2) of energy but 35.5% (SD = 18.5, SEM = 0.6) of calcium, 26.8% (SD = 19.6, SEM = 0.6) of vitamin D, 21.1% (SD = 12.5, SEM = 0.4) of phosphorus, 19.7% (SD = 15.1, SEM = 0.5) of vitamin B12, 15.8% (SD = 10.7, SEM = 0.3) of zinc, and 14.3% (SD = 11.3, SEM = 0.3) of vitamin A. Dairy products also contributed ∼23% (SD = 14.9, SEM = 0.5) of saturated fat, mostly from cheese. These observations are consistent with dietary intake data from the United States [11].

**FIGURE 6:**
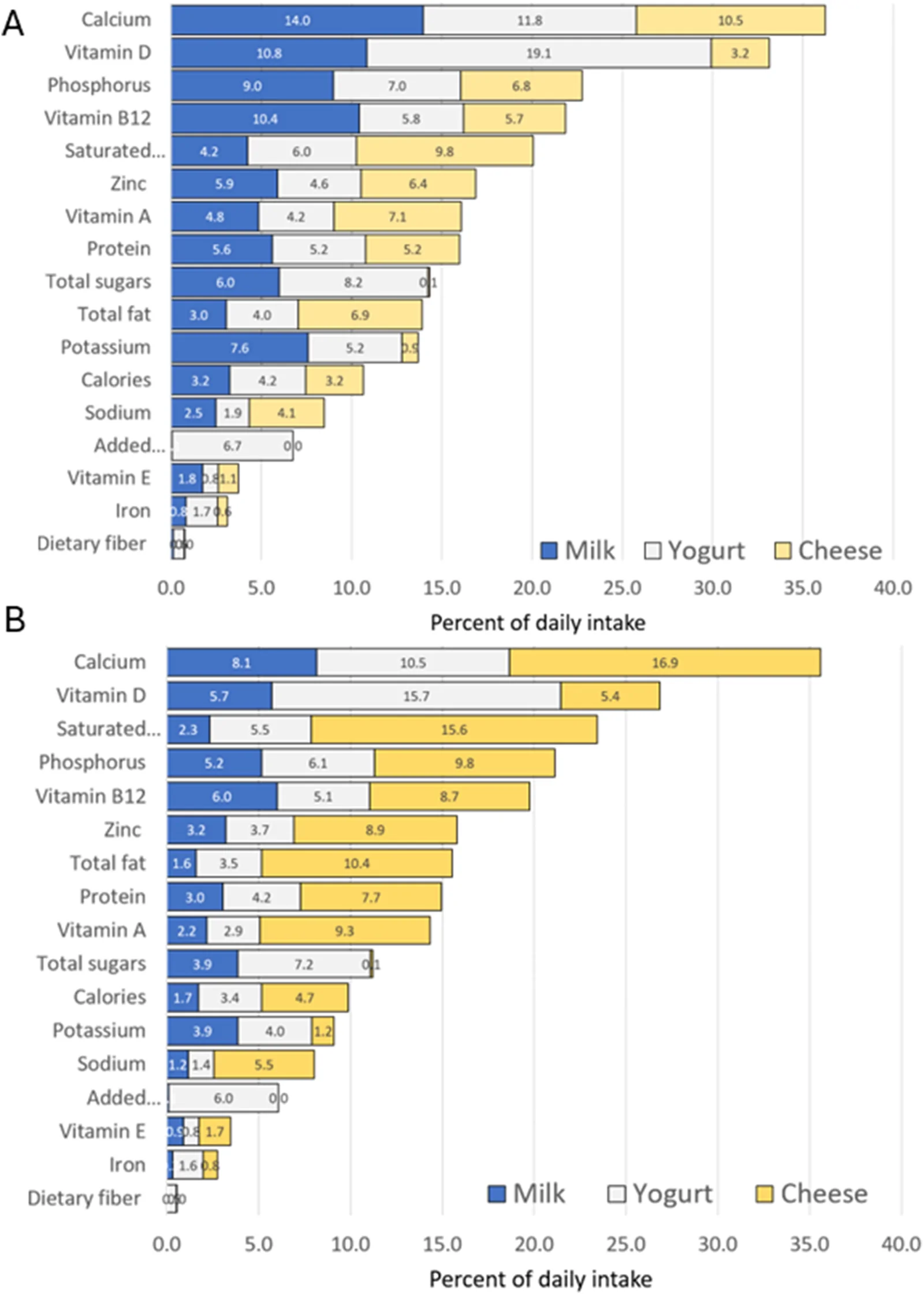
The relative contribution of milk, yogurt, and cheese to total dietary energy and selected nutrient intakes in the Étude Individuelle Nationale des Consommations Alimentaires 3 study for children (A) and for adults (B).

## Discussion

The consumption of dairy foods in France reflects a long history of agricultural production and culinary tradition. France is among the world’s top consumers of dairy products, with high reported availability of both yogurt and cheese [10,11]. Key trends in cheese consumption include a strong preference for cow milk cheeses, such as Emmental, Comté, and Camembert.

An estimated 98% of French children and adults consumed ≥1 dairy product over the 3 d of INCA3. Milk consumption was estimated at 123 g/d for children and 75 g/d for adults. Yogurt consumption was estimated at 83 g/d for children and 77 g/d for adults. Cheese consumption was estimated at 17 g/d for children and 31 g/d for adults. The total estimated intakes of milk, yogurt, and cheese were 223 g for children and 183 g for adults. These values are consistent with a previous study [38] based on the same INCA3 database.

Published estimates for dairy consumption in other European countries were very similar. Data from a national survey in Italy placed milk and dairy products consumption at 237 g/d, including 115.4 g for milk, 25.2 g for yogurt, and 37 g for cheese [39]. Data from Scotland [16] placed total intakes of dairy (including substitutes) at 238.6 g/d, with milk consumption estimated at 150 g/d. The German National Nutrition Survey II (NVS II) placed the consumption of milk, dairy products, and cheese at 259 g/d for males and 237 g/d for females [40]. By comparison, United States estimates based on NHANES data put milk consumption at 150 g/d, cheese at 34 g/d, and yogurt at only 22 g/d [41]. The consumption of fluid milk, which is strongly associated with age, has declined sharply in both France and in the United States [42].

French dietary guidelines recommend 3 daily servings of dairy for children but only 2 for adults. A serving of dairy in France is 150 mL of milk, 125-g yogurt, and 30 g of cheese [43]. The United States Dietary Guidelines recommend 3 daily servings of dairy, where a serving is 240 mL of milk, 170 g yogurt, and 30 g cheese [44]. Those guidelines are not met in the United States and would not have been met in France. The current Dietary Guidelines for Americans 2025-30 feature whole milk and butter, departing from the former recommendation to consume only low-fat dairy [45]. The French dietary guidelines continue to recommend prioritizing low-fat or reduced-fat dairy products (like semiskimmed milk and plain yogurt) over full-fat versions, primarily to limit saturated fat intake [46,47].

Published reports on the consumption patterns of milk and dairy in France [38,48] showed that fluid milk was consumed at breakfast and in the morning, especially by children and young adults. On the basis of INCA3 data for adults, yogurt and cheese were not eaten as stand-alone between-meal snacks [38]. Rather, yogurt and cheese were mostly eaten at lunch or at dinner, that is, within traditional proper meals. Interestingly, French dietary guidelines issues by PNNS (Programme National Nutrition Santé) specifically suggest consuming dairy, and notably soft dairy products (yogurt, soft cheese), as midafternoon snacks, in combination with fruits and bread [47].

Our Perspective on the high nutrient density of milk and dairy foods is supported by data from countries outside France. Data from the United States suggest that dairy products provide more nutrients than calories to the typical diet [41]. Data from Scotland [16] showed that milk products were important sources of calcium (34%), iodine (38%), and vitamin A (25%). In the INCA3 database, dairy products provided ∼10% to 12% of energy but ∼35% of the calcium intake for the French population and substantial amounts of vitamin D, phosphorus, and vitamin B12.

Milk, yogurt, and cheese provided those nutrients at a relatively low cost. Affordable nutrient-dense foods can help maintain dietary quality without increasing overall food expenditures [17,25]. Plots based on supplemental data files [19] showed that milk and dairy provided 50 g protein at relatively low cost. These data were consistent with observations in the United States that milk and dairy were the lowest-cost sources of dietary calcium by far [17] and affordable sources of high-quality protein [25].

Environmental impact is an increasingly important dimension of the sustainability framework. Interest in plant-based alternatives to milk, yogurt, and cheese continues to grow [49]. There are concerns that livestock production challenges planetary boundaries, making dairy unsustainable in the long term [4,5,8]. For example, the proposed EAT-Lancet Planetary Health Diet (1.0 version) limited dairy to 1 serving per day (250 g milk) [7]. A proposed 88% of daily calories were to come from cereals, root crops, nuts, legumes, vegetables, and fruit.

It is, therefore, important to assess both nutrient density and the environmental impact of dairy products using context-specific databases [8,50]. Studies conducted in the United States showed that the nutrient density of plant-based milk alternatives (other than soy milk) was highly variable [51]. On the basis of previously published data on environmental indicators from Agribalyse [19], dairy foods compared favorably with other animal-based foods on ≥2 environmental indicators. When compared with many meat products, dairy foods showed lower GHGE values per kg or per 100 kcal [19].

It is worth noting that the environmental cost of animal protein food sources is typically measured in terms of GHGE by weight or volume (kg or L) [51,52]. Numerous actors, including FAO committees [53] and others [19,54], have explored alternative nutrition-relevant functional units that might better serve nutrition-relevant LCAs. One proposal was to base calculations on the amount of protein (DV = 50 g/d). Alternatively, the calculations could be based on a nutrient density score as proposed by FAO [53]. The NRF scores, featured here, were one such model considered by the FAO [53].

The concept of a protein-based functional unit can be traced back to the influential article by Poore and Nemecek [55]. That article calculated GHGE values for ruminants and nonruminants and made further useful distinctions between the relative environmental impacts of beef and dairy herds [55]. However, GHGE values came from a composite list of 127 countries [55] and were not context-specific or representative of the best practices of livestock management in France. Here, the Agribalyse dataset [22] is probably the most authoritative and context-specific data source

In conclusion, health and environmental concerns related to meat production are among the main reasons behind the current initiatives to promote plant proteins on a global scale [7]. However, the concept of sustainable diets requires balancing nutritional quality, economic accessibility, cultural acceptance, and environmental impact [56]. This Perspective evaluated the place of dairy products within the French dietary context.

When assessed across the 4 pillars of sustainability—nutrition, economics, society, and environment—dairy products demonstrate strengths within each domain. Nutritionally, milk, yogurt, and cheese are nutrient-dense sources of protein and essential micronutrients. Economically, dairy products represent affordable sources of key nutrients, particularly calcium. Socially, milk, yogurt, and cheese remain deeply embedded within French culinary traditions and dietary patterns. Environmentally, dairy products compare favorably with many other animal protein sources in terms of GHGE and resource use. Milk, yogurt, and cheese represent affordable nutrient density, remain culturally embedded within French dietary traditions, and have environmental impacts that compare favorably with other food sources of animal protein.

## Author contributions

The authors’ responsibilities were as follows – AD: contributed to first draft; FV, MM: contributed to financing; and all authors: contributed to conceptualization, formal analyses, visualization, writing and editing, and all authors: read and approved the final manuscript.

## Declaration of generative AI and AI-assisted technologies in the writing process

The author(s) declare that no generative AI or AI-assisted technologies were used in the writing of this manuscript.

## Data availability

Databases described in the manuscript are publicly available without restriction from French government agencies: ANSES/CIQUAL Table de Composition Nutritionnelle des Aliments. 2016. Available online: https://ciqual.anses.fr/; Agribalyse data access. https://doc.agribalyse.fr/documentation-en/agribalyse-data/data-access; ANSES INCA3 data access: https://www.anses.fr/fr/content/inca-3-evolution-des-habitudes-et-modes-de-consommation-de-nouveaux-enjeux-en-matiere-de; Supplemental data used by Vieux et al. [19] to calculate environmental impact and cost are publicly available at: https://ars.els-cdn.com/content/image/1-s2.0-S092180092500103X-mmc1.xlsx

## Funding

Analyses of publicly available national data sets was supported by Sodiaal Dairy Cooperative, France. The sponsor had no role in study design; collection, analysis, and interpretation of data; writing of the report; or any decisions regarding the choice of journal or the submission of the report for publication.

## Conflict of interest

AD is the developer of the Naturally Nutrient-Rich and the Nutrient-Rich Food (NRF) nutrient profiling models and is or has been a member of scientific advisory panels for BEL, Lesaffre, Nestlé, FrieslandCampina Institute, National Pork Board, and Carbohydrate Quality Panel supported by Potatoes USA. He has worked with Ajinomoto, Almond Board of California, Ayanabio, FoodMinds, KraftHeinz, Lesaffre, Meiji, MS-Nutrition, Nutrition Impact LLC, Nutrition Institute, Nutrition Strategies LLC, PepsiCo, Samsung, Soremantec, and Zespri on quantitative ways to assess nutrient density of foods. FV and MM are employees of MS-Nutrition.
